# M1 and M2 macrophages markers are alternately expressed during periapical lesion development

**DOI:** 10.1590/1678-7757-2024-0579

**Published:** 2025-06-27

**Authors:** Carolina Maschietto PUCINELLI, Paulo NELSON-FILHO, Marília Pacífico LUCISANO, Jorge Esquiche LEÓN, Lúcia Helena FACCIOLI, Carlos Arterio SORGI, Clara Marina Pereira Cavalcanti SILVA, Lea Assed Bezerra da SILVA, Raquel Assed Bezerra da SILVA

**Affiliations:** 1 Universidade de São Paulo Faculdade de Odontologia de Ribeirão Preto Departamento de Clínica Infantil Ribeirão Preto SP Brasil Universidade de São Paulo, Faculdade de Odontologia de Ribeirão Preto, Departamento de Clínica Infantil, Ribeirão Preto, SP, Brasil.; 2 Universidade de São Paulo Faculdade de Odontologia de Ribeirão Preto Departamento de Estomatologia, Saúde Coletiva e Odontologia Legal Ribeirão Preto SP Brasil Universidade de São Paulo, Faculdade de Odontologia de Ribeirão Preto, Departamento de Estomatologia, Saúde Coletiva e Odontologia Legal, Ribeirão Preto, SP, Brasil.; 3 Universidade de São Paulo Faculdade de Ciências Farmacêuticas de Ribeirão Preto Departamento de Análises Clínicas, Toxicológicas e Bromatológicas Ribeirão Preto SP Brasil Universidade de São Paulo, Faculdade de Ciências Farmacêuticas de Ribeirão Preto, Departamento de Análises Clínicas, Toxicológicas e Bromatológicas, Ribeirão Preto, SP, Brasil.; 4 Universidade de São Paulo Faculdade de Filosofia, Ciências e Letras de Ribeirão Preto Departamento de Química Ribeirão Preto SP Brasil Universidade de São Paulo, Faculdade de Filosofia, Ciências e Letras de Ribeirão Preto, Departamento de Química, Ribeirão Preto, SP, Brasil.

**Keywords:** Macrophage polarization, Periapical lesion, Microscopic evaluation, Gene expression, Cytokines

## Abstract

**Background:**

This study evaluated the altered expression levels of genes and cytokines associated with M1 and M2 macrophages during the development of periapical lesion (PL).

**Methodology:**

PL was induced in the lower first molars of 96 mice. After the experimental periods of two, seven, 14, 21, and 42 days, the animals were euthanized and their jaws were dissected and submitted to the following analyses: microscopic descriptive analysis and fluorescence microscopy morphometry of PL size (mm^2^); quantitative gene expression analysis by qRT-PCR for M1 (Cxcl10, Cxcl9, and Nos2) and M2 phenotypes (Arg1, Fizz1, Ym1, and Mrc1); and M1- (GM-CSF, IFN-γ, IL-6, IL-1β, TNF-α) and M2- (IL-4, IL-13, and IL- 10) related cytokines quantification by Luminex. Data were statistically compared by ANOVA, Tukey post-test, Kruskal-Wallis, and Dunn post-test (α=5%).

**Results:**

PL area and inflammatory infiltrate increased over experimental periods. From a contextual view, a pro-inflammatory profile and a higher activation of M1 phenotype markers in the initial periods of two and seven days could be observed. On day 21, microscopic features and M2 subtype predominance indicated a repair attempt. However, on day 42, an acute exacerbation of immunoinflammatory process and return to the M1 macrophage profile were evidenced.

**Conclusion:**

M1 and M2 macrophage polarization-related markers were expressed alternately throughout the experimental periods, according to the stage of PL progression.

## Graphical abstract

**Figure:**
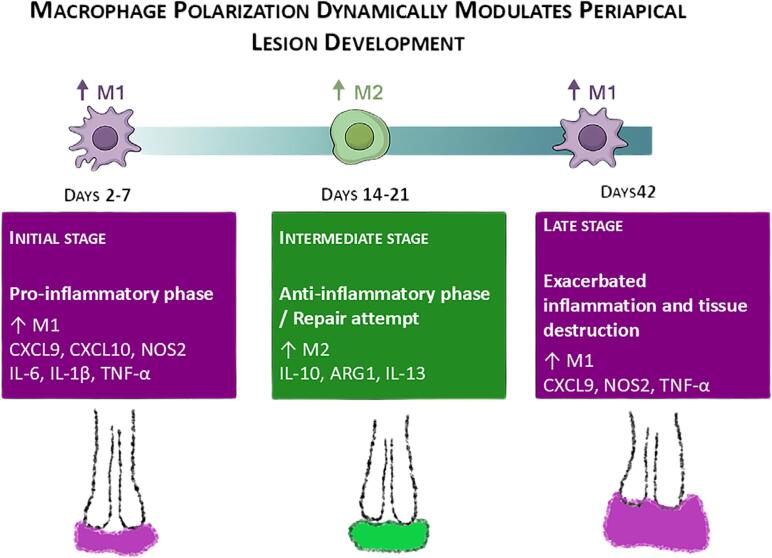

## Introduction

The inflammatory immune response underlying the pathogenic mechanisms of endodontic lesions involves an orchestrated interplay of multiple cell types and a branched cytokine network.^1-3^ Development, progression, and outcome of periapical lesions (PLs) are determined by the complex interaction between pathogens and the host immune system.^4,5^

Macrophages are essential cells for the entire process and key activators of innate immune responses by recognition of pathogens, in addition to mediating adaptive arm of immunity.^3,5-7^ At different stages of the inflammatory response, macrophages can have mainly two subtypes, classically (M1) or alternatively (M2) polarization.^8^ Dependent on the microenvironment to which monocytes and macrophage precursor cells are exposed during their differentiation, such cells could be polarized into the different phenotypes.^9^

Some studies have shown the relationship between macrophage polarization and development of PLs.^7,10,11^ Classical macrophage activation (M1 macrophage) is stimulated by bacterial lipopolysaccharides (LPS), Granulocyte and Macrophage Colony Stimulating Factor (GM-CSF), and Interferon gamma (IFN-γ). Once the infection is detected, M1 macrophages are responsible for the response activation of the host^9,12,13^ and then initiate a significant cytotoxic function against infected cells,^14,15^ especially secreting reactive oxygen and nitrogen species, inducible nitric oxide synthase (iNOS), and promoting arginine metabolism.^9,12^ In contrast, M2 subtypes are essential for tissue repair,^9^ acting on remodeling and angiogenesis processes.^16,17^

Then, macrophages cells play a significant role in both the destructive and reparative phases of periodontal disease.^18,19^ During the early stages of periodontitis, these cells differentiate into M1-subtype and secrete high levels of pro-inflammatory cytokines and chemokines, such as IL-1β, IL-6, TNF-α, IFN-γ,^5,10,20^CXCL9, and CXCL10. As the disease progresses into its restorative phase, the proportion of M2-subtype macrophages increases, in which anti-inflammatory and T helper 2 (Th2) cytokines are released, such as IL-4, IL-10, and IL-13.^9,21^ According to Song, et al.^5^ (2022), the spatial and temporal expression profile of macrophages might reflect the status of PLs.

Although emerging evidence has demonstrated the association of macrophage M1 and M2 subpopulations with the immunoinflammatory state of chronic endodontic lesions,^7,10,20,22^ the precise description of M1 and M2 participation in pathogenesis of PL, as well as their involved signaling pathways, still needs to be fully understood. Considering the critical role in the orchestration of PL progression, the knowledge of participation and regulatory networks underlying macrophage polarization sheds light on the clinical diagnosis and potential therapeutic targets. Then, this study aimed to evaluate the phenotypic characterization of M1 and M2 macrophage subtypes during the development of induced apical periodontitis in mice teeth.

## Methodology

The design of this study was based on the Animal Research Reporting *In Vivo* Experiments (ARRIVE) guidelines,^23^and the procedures were previously approved by the Institutional Animal Use Ethics Committee (2016.1.645.58.0).

Ninety-six male C57BL/6 mice, from six to eight weeks old and weighing 20 grams, were used. They were kept at an animal facility with free access to food and water. Firstly, the mice were anesthetized with ketamine (150 mg/kg; AGENER S/A, Brazil) and xylazine (7.5 mg/kg; DOPASER, Spain) and were positioned on a restraint table for periapical lesion induction.^24-27^ The pulpal tissue was exposed to the oral microenvironment through a cavity created in the tooth crown, using an electric handpiece (DABI ATLANTE, Brazil) and a low-speed round drill (¼, KG SORENSEN, Brazil).

The PL was induced in both mandibular first molars of 80 animals (experimental group), while the other 16 animals were the control group (sound teeth, without apical periodontitis). The animals were randomly allocated to each group (Experimental and Control groups), and each tooth constituted an experimental unit.

After the experimental periods of two, seven, 14, 21, and 42 days, the animals were anesthetized and euthanized,^25-27^ and the jaws were dissected for further processing and analyses.

An experimental design flowchart is showed to clarify the design and arrangement of groups, as follows:

Total Animals (n=96)

→Control Group (n=16 animals)

• Healthy teeth
• Right Molars of eight animals →Histological analysis (n=8 teeth)
• Left Molars of the same eight animals →qRT-PCR (n=8 teeth)
• Right Molars of the remaining eight animals →Cytokine Quantification (Luminex) (n=8 teeth).

→Experimental Group (n=80 animals)

• Periapical Lesion induced
• Divided into five time-points:
- two, seven, 14, 21, and 42 days
- n=8 teeth per time-point per analysis
• Right Molars of 40 animals →Histological analysis (n=8 teeth per time-point)
• Left Molars of the same 40 animals →qRT-PCR (n=8 teeth per time-point)
• Right Molars of the remaining 40 animals →Cytokine Quantification (Luminex) (n=8 teeth per time-point).

### Histotechnical processing and microscopic evaluation

For hematoxylin and eosin (HE) microscopic analysis and fluorescence microscopy morphometry, 40 animals with induced PL (n=8 teeth/experimental period) and eight control animals (n=8 sound teeth for the control group) were used.

After euthanasia, the right hemi-jaws were removed and fixed in formaldehyde. Then, the specimens were subjected to a histotechnical process and embedded in paraffin. Semi-serial 5μm sections were obtained and stained with HE.

Then, the sections were subjected to descriptive analysis of pulp, apical, and periapical tissues by conventional optical microscopy.

**Figure 1:**
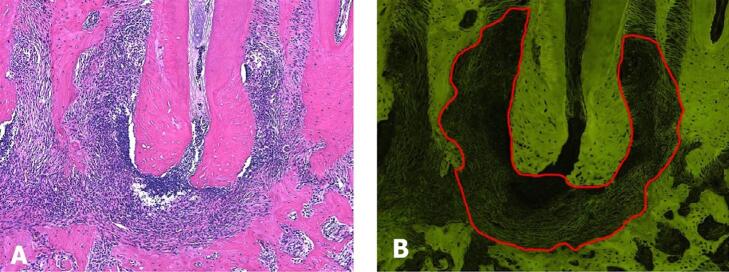
Representative image of morphometry in fluorescence microscopy. In (A), the PL microscopic characteristics in HE are observed. In B, the same HE-stained section in fluorescence mode is observed, highlighting the delimitation of the PL area. Images at 100 X magnification.

In parallel, HE-stained slides were evaluated in fluorescence mode, at a 10X objective.^24,25,27^ The periapical lesion area was outlined and measured in mm^2^, excluding the intact structures and including areas of resorption and inflammatory infiltrate (Figure 1).

All analyses were performed by a single-blinded experienced and calibrated evaluator, using an Axio Imager. M1 microscope (Carl Zeiss MicroImaging GmbH, Göttingen, Germany) coupled with an AxioCam MRc5 camera (Carl Zeiss MicroImaging GmbH, Göttingen, Germany).

### Gene expression analysis - qRT-PCR

For the gene expression analysis, the left hemi-jaws of the same 40 animals with induced PL (n=8 teeth/experimental period) and eight control animals (n=8 sound teeth) were used.

For the qRT-PCR protocol, the specimens were subjected to the ribonucleic acid (RNA) extraction process, according to the manufacturer’s recommendations of the PureLink™ RNA kit (Ambion^®^, Life Technologies, Carlsbad, CA, USA) and the extracted RNA was estimated with NanoDrop One (Thermo Fisher Scientific Inc., Wilmington, DE). From 1µg of total RNA, the complementary DNA strand (cDNA) was made by a reverse transcription reaction based on the manufacturer protocol using the High-Capacity kit (Applied Biosystems, Foster City, California, USA). The amplification reaction of each gene consisted of cDNA-specific primers, the TaqMan fluorescence system (Applied Biosystems^®^, Foster City, CA, USA) and MasterMix reagent (Applied Biosystems). Glyceraldehyde-3-phosphate dehydrogenase (Gapdh) and β-actin (Actb) gene expression were analyzed as housekeeping genes. Cxcl10, Cxcl9, and Nos2 mRNA (for M1 phenotype) and Arg1, Ym1, Fizz1, and MRC1 (for M2 phenotype) were evaluated. Quantitative analysis of mRNA expression was performed using the StepOnePlus™ Real-Time PCR System (Applied Biosystems^®^, Foster City, CA, USA), under conditions of 95°C (two minutes), followed by 40 cycles of 95°C (one second) and 60°C (20 seconds). The reactions were performed in duplicate for each sample. After sample amplification, the relative quantification was performed using the 2-ΔΔCt method. The relative expressions of the evaluated genes of the groups of teeth with PLs were normalized by their respective sound controls at each experimental time interval (two, seven, 14, 21, and 42 days).

### Cytokine quantification using luminex®

For the cytokine quantification via Luminex^®^ assay, the right hemi-jaws of the remaining 40 animals with induced PL (n=8 teeth/experimental period) and eight control animals (n=8 sound teeth/control group) were used. The Millipore 7-plex kit (Millipore Corporation, Billerica, MA, USA) was used to determine cytokine concentrations. Initially, the hemi-mandibles were eluted with 60μL of buffer from the kit, placed in a vortex for 30 minutes and centrifuged twice (10 minutes at 10,000 rpm). The kit enabled the measurement of: GM-CSF, IFN-γ, IL-4, IL-13, IL-10, IL-6, IL-1β, and TNF-α.

The assays were performed in 96-well plates with a filtration membrane at their base, following the manufacturer’s instructions. Microspheres coated with monoclonal antibodies against the cytokines analyzed were added to the wells of the pre-moistened plate. The samples and standards (between 0.13 and 2000 pg/mL for each analysis) were pipetted and incubated overnight at 4°C. The wells were washed with a vacuum manifold (Millipore Corporation) and a mixture of biotinylated secondary antibodies was added. After incubation for one hour, streptavidin conjugated to the fluorescent protein R-phycoerythrin (streptavidin-PE) was added to the beads and the set was incubated at room temperature for 30 minutes. After washing to remove non-adhered reagents, a sheath fluid buffer solution (Luminex^®^, MiraiBio, Alameda, CA) was added to the wells for evaluation on a microsphere analyzer (Luminex^®^ 100TM, Luminex^®^, MiraiBio, Alameda, CA) to determine the amount of fluorescence emitted by the microspheres associated with phycoerythrin, reported as the mean fluorescence intensity.

Sample concentrations were quantified from the standard curve using a third-order polynomial equation on GraphPadPrism 5 software (GraphPad Software Inc., La Jolla, CA, USA), with values expressed in pg/mL. For each sample, the normalization was performed using the results obtained from the Luminex^®^ assay divided by the total amount of protein. BCA Protein Assay Kit (Novagen - Darmstadt, Germany) was used according to manufacturing protocol for the total protein quantification. The samples were measured using a spectrophotometer SpectraMax Paradigm Multi-Mode Microplate Reader (Molecular Devices, LLC., San Jose, CA, EUA) at 562 nm wavelength

Each cytokine evaluated from the PL group was normalized by their respective sound controls at each experimental time interval (two, seven, 14, 21, and 42 days).

### Statistical analysis

The data were subject to the Shapiro-Wilk normality test and were found to be normally distributed. Results of PL size were compared by ANOVA test and Tukey post-test. For the values obtained from RT-PCR and Luminex^®^ assay, the Kruskal-Wallis test and Dunn post-test were used. The significance level adopted was 5%. All analyses and graphical representations were performed using GraphPad Prism 7a Software (GraphPad Software Inc., San Diego, CA, USA).

## Results

### Microscopic evaluation

The control group (sound teeth) showed periodontal ligament, bone, and apical and periapical tissues with aspects of normality. There was an absence of inflammatory infiltrate and resorption of mineralized tissues.

After day two of PL induction, the periodontal area was slightly enlarged, with mild lymphomononuclear inflammatory infiltrate, supported by a delicate fibro-vascular stroma. On day seven, the PL progressed with an increase of periodontal ligament. The fibro cellular stroma was permeated by a scattered lymphomononuclear inflammatory infiltrate, ranging from mild to moderate.

Fourteen days after PL induction, a more intense inflammatory infiltrate was observed, with a significant increase of the periodontal ligament space. The specimens showed areas of poorly defined basophilic tissue and liquid accumulation (edema), which were compatible with tissue necrosis process. On day 21, there was no increase in the periodontal ligament and the fibro cellular stroma was permeated by sparce lymphomononuclear cells. In addition, a membranous neoformation process was observed, with bone trabeculae in the early stages of mineralization, which indicates a repair attempt. Nonetheless, after 42 days, the persistence and maintenance of the inflammatory process in the periapical region at a more advanced stage was observed, with a very intense inflammatory infiltrate throughout the PL. The fibro cellular stroma was less evident and the lesion was entirely permeated by edema and fibrillar dissociation, with intense bone and root resorption.

**Figure 2:**
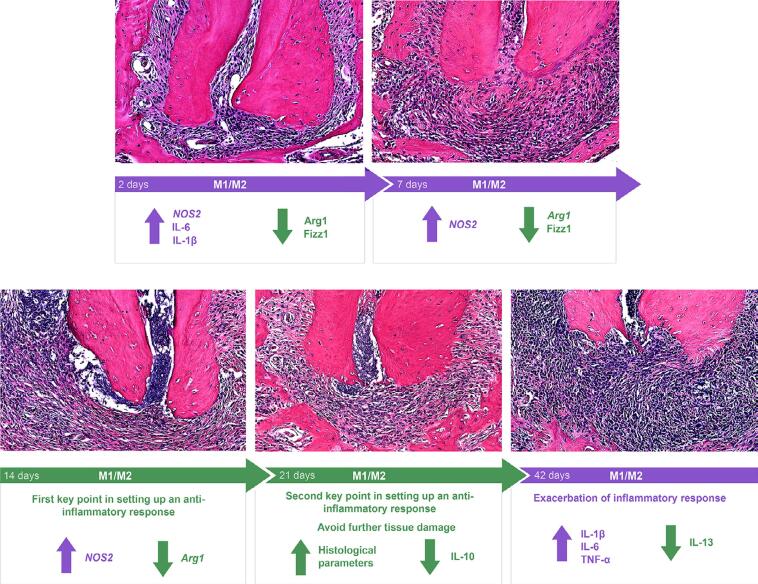
Periapical lesion progression at two, seven, 14, 21, and 42 days: data from microscopic, qRT-PCR and Luminex®. Arrows represent an increase or decrease in expression levels. Purple indicates markers related to M1 macrophage, whereas green indicates markers associated with M2 macrophage.

Figure 2 shows the described microscopic features of PL progression at two, seven, 14, 21 and 42 days.

### Fluorescence microscopy morphometry

**Figure 3:**
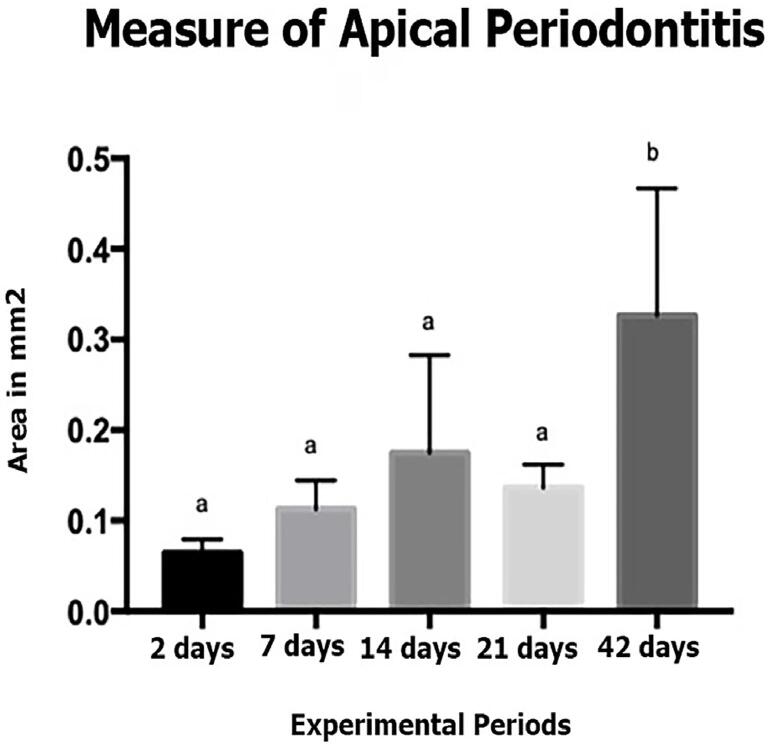
Graphical representation of the PL area measurements at two, seven, 14, 21, and 42 days. Values are expressed in mm2. Different letters mean a statistically significant difference (p<0.05).

The morphometric assessment of the periapical lesion area showed a statistically significant difference between experimental periods (p<0.05). The PL size at 42 days was significantly higher compared to other periods (two, seven, 14, and 21 days) (p<0.05). Figure 3 graphically demonstrates the comparison between the fluorescence microscopy-based periapical lesion area measurements at different experimental periods.

### Gene expression analysis – qRT-PCR

#### Markers of M1 macrophage phenotype (Cxcl10, Cxcl9, and Nos2)

On day two after PL induction there was the lowest level of Cxcl10 expression. Such values increased significantly on day seven, pointed as the expression peak of Cxcl10. When experimental periods were compared, the two day-time point was statistically different from seven and 42 days (p=0.0042). Initially, there was the lowest level of Cxcl9 expression, which progressively increased with PL development, showing its expression peak on day 42. When experimental periods were compared, a statistical difference was observed between two days, and 14, 21, and 42 days (p=0.0001). In addition, the seven-day values were statistically different from the 42-day period (p=0.0001). Regarding Nos2, the highest expression was on day two, followed by a statistically significant reduction at seven days (p=0.0311). On days seven and 21 there was a decrease of Nos2 expression, and an increase at days 14 and 42. However, a statistical difference among these experimental periods was not observed (p˃0.05).

#### Markers of M2 macrophage phenotype (Arg1, Fizz1, Ym1, and Mrc1)

Day two showed a significantly higher expression of Arg1 when compared to day seven (p=0.014), which showed the lowest values. There was the expression peak of Arg1 on day 14, which was statistically different from day seven (p=0.0049). Compared to day 2, the values of Arg1 expression decreased on days 21 and 42. In general, there was no statistically significant difference among the PL experimental periods regarding the Fizz1 expression (p˃0.05), although the periods of 14 and 21 days had the highest values. The expression peak of Ym1 was on day seven, being statistically different from day 14 (p=0.002), which had the lowest values. Compared to the 14-day period, there was a significant increase on day 21 (p=0.002), with stabilization of this expression at 42 days. Finally, no statistically significant difference was observed among the experimental periods of PL regarding Mrc1 expression (p˃0.05), although day 42 has shown the highest values.

Figure 2 shows graphical representations of the relative expression ratio of the evaluated genes.

**Figure 5:**
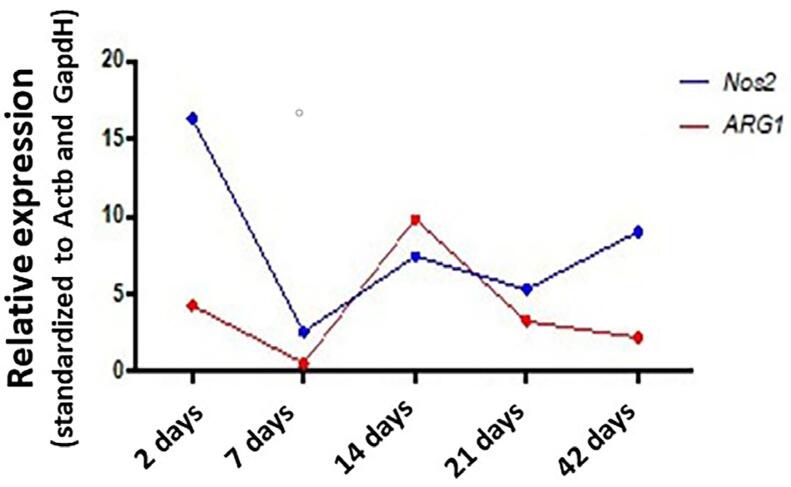
Graphical representation of Nos2 and Arg1 relative expression, indicating M1 and M2 macrophage phenotypes throughout the PL development (two, seven, 14, 21, and 42 days).

*Nos2* and *Arg1* are well-established markers for the M1 and M2 macrophage phenotype analysis, respectively. Figure 5 illustrates the comparison of *Nos2* and *Arg1* expression levels along the experimental periods of periapical lesion development. The overall analysis of the gene expression results indicated a more intense activation of M1 phenotype macrophages in almost all evaluated time points. The first activation peak occurred on day two and a second one occurred on day 42. The lowest activation of M1 macrophage was observed on day seven. Interestingly, over 14 days, M1 expression decreased and was overcome by the expression of M2 macrophage marker (Figure 5).

**Figure 4:**
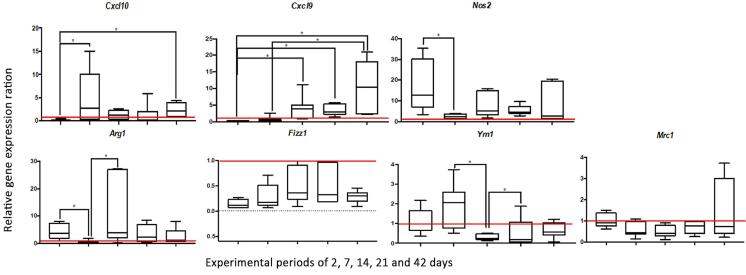
Graphical representations of the relative expression ratio of the evaluated genes for both M1 (Cxcl10, Cxcl9, and Nos2) and M2 (Arg1, Ym1, Fizz1, and MRC1) macrophage phenotypes. The values of specimens with periapical lesions in periods of two, seven, 14, 21, and 42 days were normalized with the values of healthy teeth in the control group (indicated by the horizontal line). * indicates statistically significant difference.

## Cytokine quantification

### Markers of M1 macrophage phenotype (GM-CSF, IFN-γ, IL-6, IL-1β, TNF-α)

The data showed an increase in GM-CSF overtime, with the lowest value on day two and a peak of expression on day 21. When the experimental periods were compared, day two showed statistical difference from days 14, 21, and 42 (p=0.001). The lowest IFN-y values were observed on day seven, while the highest amounts occurred on days 14 and 21. Then, statistically significant differences were observed between day seven compared with days 14 and 21 (p=0.002). Initially, PL on day two showed high levels of IL-6, that reduced after seven and 14 days, in which the last showed the lowest values. After 21 and 42 days of PL progression, IL-6 expression increased again. The IL-6 expression on day 14 was statistically different in comparison to the values obtained on days two and 21 (p=0.002). Similarly, the highest amount of IL-1β was observed on day two, decreasing on days seven and 14, and returning to higher values on days 21 and 42. There was a statistically significant difference between days two and 14 (p=0.001). Also, days seven and 14 were statistically different from day 42 (p=0.001), which showed a higher amount of IL-1β. Finally, TNF-α levels increased during the progression of PL, reaching a peak in periods of 21 and 42 days. The values on days two and 14 were statistically lower than those on day 42 (p=0.001).

### Markers of M2 macrophage phenotype (IL-4, IL-13, and IL- 10)

In the initial periods of two and seven days of PL, higher values of IL-4 expression were observed, which were statistically reduced to the lowest levels in the 14-day period (p=0.017). After 21 and 42 days, the amount of IL-4 increased progressively again, showing a statistical difference between days 42 and 14 (p<0.05). Basal levels of IL-13 were detected at two, seven and 14 days of PL. The IL-13 expression was progressively higher on days 21 and 42, making it possible to find the following statistical differences: days two and 14 compared to day 42; and day seven compared to days 21 and 42 (p<0.0001). Finally, the analysis of IL-10 expression showed the lowest values on day 14 and the highest on day 21. Then, the periods of seven and 14 days showed statistically lower levels of IL-10 compared to those of 21 days (p=0.0002).

**Figure 6:**
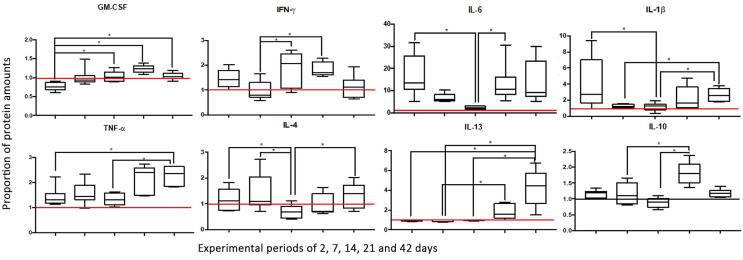
Graphical representations of the proportion of protein amounts for both M1 (GM-CSF, IFN-γ, IL-6, IL-1β, and TNF-α) and M2 (IL-4, IL-13, and IL-10) macrophage phenotypes. The values of specimens with periapical lesions in periods of two, seven, 14, 21 and 42 days were normalized with the values of healthy teeth in the control group (indicated by the horizontal line). * indicates statistically significant difference.

Figure 6 shows graphical representations of the proportion of protein amounts.

In addition, Figure 2 summarizes the main findings of gene and protein expression of M1 and M2 macrophage subtypes and their correlation with microscopic overview of PL development in each period. Overall, a pro-inflammatory profile and a higher activation of M1 phenotype markers in the initial periods of two and seven days were observed, which started to reverse after 14 days. On day 21, there is evidence of a repair attempt, with an anti-inflammatory response and a predominance of the M2 subtype. However, on day 42 and persistence of the infections, an acute exacerbation of immunoinflammatory process is observed, returning to the M1 macrophage profile of PL.

## Discussion

The importance of understanding the role of macrophage phenotypes in different pathologies and their use as a therapeutic target have been highlighted in the literature.^17,28,29^ However, the absence of studies evaluating microscopic, molecular, and gene expression aspects involved in macrophage polarization during the progression of periapical lesions (PLs) led to this study’s aim.

Our results showed that the PL progression occurred dynamically, and markers for both M1 and M2 macrophages were present at different levels throughout the experimental periods. Macrophages have a gene expression profile that is influenced by the type, concentration, and duration of exposure to the stimulating factors.^30^ Understanding the inflammatory microenvironment is fundamental to study macrophage polarization in M1 and M2 subtypes. Then, cytokines, chemokines, and growth factors related to macrophage activation and molecules expressed after their polarization were evaluated.

The chemokines CXCL9, CXCL10, and CXCL11 are potent chemoattractants of mononuclear cells^31^ and play key roles to induce T helper 1 (Th1) cells differentiation,^32^ being related to M1 macrophage polarization.^33^ This study demonstrated that the Cxcl9 expression increased progressively over time, with a peak on day 42. However, Cxcl10 was significantly expressed on day seven, with smaller amounts on day 42. Considering other markers for the M1 subtype, Nos2, IL-6, IL-1β, and TNF-α showed considerable expression on days two and 42 of PL.

To study the phenotypic polarization of macrophages, it is necessary to understand the inflammatory microenvironment in which these macrophages are inserted. On day seven, Cxcl10 may be functioning as a chemotactic factor for macrophages. However, on day 42, the inflammation shows a different profile. Cxcl9, Cxcl10, and Nos2 gene expressions are increased, as well as protein markers for M1, such as IL-6, IL-1β and TNF-α.

The chemotactic agents CXCL9 and CXCL10 attract cells to the infection site, which secrete more pro-inflammatory cytokines, such as TNF-α, IL-1β and IFN-γ, amplifying the process and exacerbating the Th1 response. It is well established that Th1 response cytokines TNF-α and IL-1β are potent inducers of osteoclastogenesis and bone resorption.^34,35^ Such molecular signals impact the larger tissue response evidenced at the final period of PL.

In addition, IL-6 has been considered a mediator of periapical bone loss^36^ and its levels are proportional to the PL size.^37^ Also, IL-6 has been described as having a pleiotropic characteristic^35^, sometimes pro-inflammatory and sometimes anti-inflammatory, with its effect modulated by the local immune microenvironment, target cells and the interaction with other cytokines.^38^ Thus, the increase in IL-6 correlates directly with the observed bone resorption and global features in the later stages of PL.

In line with the results of this study, nitric oxide synthase (NOS2), a marker tightly connected with M1-subtype macrophage polarization,^8^ is induced by TNF-α, IL-1, IFN-γ, and LPS.^39^NOS2 is related to the production of NO in macrophages, associated with oxidative stress and local tissue destruction.^40^

This response pattern can be correlated with the microscopic features of PL development. Initially, a pro-inflammatory microenvironment is established to eliminate the endodontic pathogens, but which returns to predominate in the final period (day 42) with the persistence of the infectious stimulus.

Since the root canals were not treated, the bacterial etiological agents were not removed and therefore the inflammatory stimulus remained. Then, despite the organism’s attempt to control the immunoinflammatory process, on day 42, there was an acute exacerbation of inflammation, with great tissue destruction and cellular necrosis, and an increase in pro-inflammatory markers and differentiation of macrophages to the M1 phenotype, such as Cxcl9, Nos2, GM-CSF, IL-1β and TNF-α. During the progression of PL, the host attempted to establish an anti-inflammatory response. On day 14, there was an increased expression of Arg1 (arginase 1), an anti-inflammatory cytokine released by M2 macrophages. This subtype is classically activated by IL-4 and IL-10.^41^ Interestingly, the development of the M2 profile at a later stage is associated with the T helper 2 (Th2)/Tregs axis activation and arrest of disease progression.^42,43^ Also, Th2 related cytokines IL-4 and 1L-13 skew macrophage polarization into the M2 phenotype.^44,45^

A recent study explored the role of efferocytosis, a process in which the apoptotic neutrophils in diseased tissues are engulfed by macrophages, in the pathogenesis of apical periodontitis.^46^ In line with our perspective, the *in vivo* and *in vitro* results suggested that macrophage efferocytosis promotes the resolution of apical periodontitis by accelerating M2 subtype polarization.^46^

The findings demonstrated a higher IL-4 expression on day seven, which can be suggested that this cytokine stimulated M2 polarization, since Arg 1 is strongly regulated by IL-4.^39^ Interestingly, IL-4 was previously associated with an antiosteoclastogenic action and a possible protector effect on PL activity.^47^ Another Th2 related cytokine IL-10 had its peak expression on day 21, a period associated with the repair attempt and predominance of M2 macrophages. Confirming its potential suppressive function, *Knockout* mice for IL-10 developed greater PLs.^48^ Microscopically, on day 21 the periodontal ligament space did not increase and there was the presence of newly formed bone tissue. In partial agreement, considerable amount of IL-13 was only detected at later periods of PL progression (days 21 and 42).

Mannose receptor C-type 1 (Mrc1) is an indicator of M2 macrophage activation, and its expression is induced by IL-13. In this study, the 42-day experimental period showed a numerically higher quantity of Mrc1, coinciding with the significant increase in IL-13.

Although IFN-γ is a classic marker for M1 profile and may have had a dual effect on osteoclasts, this cytokine has demonstrated a role in inhibiting osteoclast activation and promoting bone maintenance.^49^ In agreement, according to De Rossi et al.^48^ (2008), IFN-γ may have a protective effect on periapical bone resorption. In this line and following our results, the highest IFN-γ expression was observed on the days 14 and 21. Additionaly, GM-CSF had its higher expression on day 21, because it is related to the growth and differentiation of cells.^50^ Them, its increase at 21 days can be justified by greater proliferative and formative activity in the context of the PL.

According to Locati, Curtale and Matovani, et al.^20^(2020), translating the understanding of the phenotypic and functional diversity of macrophages into clinically useful signatures and biomarkers remains a challenge. PL development involves a dynamic and complex imunoinflammatory response, controlled by an extensive network of several mechanisms, including the release of cytokines, the production of chemotactic molecules, and the expression of cell surface adhesion molecules.^48^ This study evidenced this highly coordinated and orchestrated process, with compensatory pathways and mechanisms activation.

It is important to highlight some limitations inherent to the study model and methodologies. Whereas murines are valuable for studying the mechanisms underlying immune-mediated periapical pathology, further validation using complementary experimental models is essential for direct translational clinical applicability. The qRT-PCR and Luminex techniques were performed on dental tissue blocks (tooth, alveolar bone, and periodontal ligament) and therefore did not provide the exact number of markers in the periapical lesion nor directly identify the producing cell. Furthermore, a distinct spatial localization of M1 and M2 macrophages in the lesion sites that would correspond with tissue destruction or repair events was limited.

In addition to the fact that cells may express both M1- and M2-associated markers simultaneously depending on the microenvironmental stimuli,^51^ markers commonly used to define these subtypes (e.g., iNOS for M1; Arg1 for M2) can be co-expressed or upregulated in non-macrophage populations, complicating data interpretation.

In conclusion, M1 and M2 macrophage polarization-related markers were expressed alternately throughout the experimental periods, according to the stage of PL progression. In the early and final time points, there was a predominance of M1 phenotype, compatible with the establishment of a pro-inflammatory microenvironment and disease activity. Nonetheless, at intermediate stages, more specifically on days 14 and 21, a repair attempt was microscopically and molecularly evidenced, showing an anti-inflammatory profile and M2 subtype predominance.
